# Progress Toward Global Eradication of Dracunculiasis — January 2018–June 2019

**DOI:** 10.15585/mmwr.mm6843a5

**Published:** 2019-11-01

**Authors:** Donald R. Hopkins, Adam J. Weiss, Sharon L. Roy, James Zingeser, Sarah Anne J. Guagliardo

**Affiliations:** ^1^The Carter Center, Atlanta, Georgia; ^2^Division of Parasitic Diseases and Malaria, Center for Global Health, World Health Organization Collaborating Center for Dracunculiasis Eradication, CDC.

Dracunculiasis (also known as Guinea worm disease) is caused by the parasite *Dracunculus medinensis* and is acquired by drinking water containing copepods (water fleas) infected with *D. medinensis* larvae. The worm typically emerges through the skin on a lower limb approximately 1 year after infection, resulting in pain and disability (*1*). There is no vaccine or medicine to treat the disease; eradication efforts rely on case containment* to prevent water contamination and other interventions to prevent infection, including health education, water filtration, chemical treatment of unsafe water with temephos (an organophosphate larvicide to kill copepods), and provision of safe drinking water (*1*,*2*). In 1986, with an estimated 3.5 million cases^†^ occurring each year in 20 African and Asian countries^§^ (*3*), the World Health Assembly called for dracunculiasis elimination (*4*). The global Guinea Worm Eradication Program (GWEP), led by The Carter Center and supported by the World Health Organization (WHO), CDC, the United Nations Children’s Fund, and other partners, began assisting ministries of health in countries with dracunculiasis. This report, based on updated health ministry data, describes progress to eradicate dracunculiasis during January 2018–June 2019 and updates previous reports (*2*,*4*,*5*). With only five countries currently affected by dracunculiasis (Angola, Chad, Ethiopia, Mali, and South Sudan), achievement of eradication is within reach, but it is challenged by civil unrest, insecurity, and lingering epidemiologic and zoologic questions.

In March 2018 and March 2019, The Carter Center hosted the annual GWEP managers meetings in Atlanta, Georgia. WHO’s International Commission for the Certification of Dracunculiasis Eradication met in Addis Ababa, Ethiopia, in April 2019, and WHO convened the annual informal meetings of Ministers of Health of current and former endemic dracunculiasis countries during the World Health Assemblies in Geneva, Switzerland, in May 2018 and May 2019. WHO has certified 199 countries, areas, and territories as free from dracunculiasis (*4*); seven countries still lack certification: four with endemic dracunculiasis (Chad, Ethiopia, Mali, and South Sudan), one in the precertification stage (Sudan), and two that were never known to have endemic dracunculiasis (Angola and the Democratic Republic of Congo). While preparing for certification, Angola discovered a case of dracunculiasis in 2018.

In 2018, 28 indigenous human cases were reported from Angola, Chad, and South Sudan, and 1,102 infected animals (mostly dogs) were reported from Chad, Ethiopia, and Mali, compared with 30 human cases and 855 animal infections reported in 2017 (Table 1). During January–June 2019, human cases were reported in Chad (23 cases), Angola (one), and Cameroon (one), with 1,345 infected animals reported, compared with nine human cases and 709 infected animals reported during January–June 2018. During January–June 2019, CDC received 39 specimens from humans, including 16 (41%) that were laboratory-confirmed as *D. medinensis*,^¶^ compared with 89 specimens received and 38 (43%) confirmed during all of 2018. (Table 2). During the first 6 months of 2019, CDC received seven specimens from animals, five (31%) of which were confirmed, compared with 13 received and nine (18%) confirmed during 2018. *D. medinensis* worms removed from animals are genetically indistinguishable from those removed from humans (*6*).

**TABLE 1:** Number of reported indigenous dracunculiasis cases, by country –– worldwide, January 2017–June 2019

Country	No. of cases (% contained)		% change in no. of cases, Jan–Dec 2017 to Jan–Dec 2018	No. of cases (% contained)		% change in no. of cases, Jan–Jun 2018 to Jan–Jun 2019
	Jan–Dec 2017	Jan–Dec 2018		Jan–Jun 2018	Jan–Jun 2019	
**Human cases**						
Chad	15 (67)	17 (41)	+13	4 (100)	23 (61)	+475
Ethiopia	15 (20)	0	−100	0	0	0
Mali*	0	0	0	0	0	0
South Sudan	0	10 (30)	NA	4 (0)	0	−100
Angola	0	1 (0)	NA	1 (0)	1 (0)	0
Cameroon^†^	0	0	0	0	1 (0)	NA
**Total**	**30 (43)**	**28 (36)**	**−7**	**9 (44)**	**25 (56)**	**+178**
**Animal infections^§,¶^**						
Chad	830 (75)	1,065 (75)	+28	696 (74)	1,356 (78)	+95
Ethiopia	15 (40)	17 (41)	+13	10 (70)	6 (0)	−40
Mali*	10 (80)	20 (80)	+100	3 (67)	0	−100
Angola	0	0	0	0	1 (0)	NA
**Total**	**855 (75)**	**1,102 (75)**	**+29**	**709 (74)**	**1,363 (78)**	**+92**

**Abbreviation:** NA = not applicable.

* Civil unrest and insecurity resulting from a coup d’état in April 2012 continued to constrain program operations in regions with endemic dracunculiasis (Gao, Kidal, Mopti, and Timbuktu) during January 2017–June 2019.

^†^ Final classification of case origin pending further investigation.

^§^ In Chad, primarily dogs, some cats; in Ethiopia, dogs, cats, and baboons; in Mali, dogs and cats; in Angola, one dog.

^¶^ No international importations of animal infections were reported during the 18-month period January 2018–June 2019.

**TABLE 2:** Characteristics of specimens from humans and animals received at CDC for laboratory diagnosis of *Dracunculus medinensis* — January 2018–June 2019

Specimens received at CDC	Jan–Dec 2018	Jan–Jun 2019
**Specimens from humans**		
No. received	89	39
No. laboratory-confirmed as *Dracunculus medinensis* (%)	38 (43)	16 (41)
**Country of origin, no. of specimens (no. of patients)**		
Angola	1 (1)	1 (1)
Chad	21 (17)*	15 (15)**^†^**
Ethiopia	1 (1)^§^	—
South Sudan	15 (10)	—
**No. ruled out as *D. medinensis* (%)**	51 (57)	23 (59)
**No. of other laboratory diagnoses (%)**		
Mermithid	7 (14)	1 (4)
*Onchocerca*	5 (10)	2 (9)
Sparganum	5 (10)	10 (43)
Earthworm	3 (6)	—
*Dirofilaria*	1 (2)	—
Ascarid	1 (2)	—
*Eleaophora*	—	1 (4)
Worm of unknown species	7 (14)	1 (4)
Connective tissue	13 (25)	6 (26)
Unknown origin	9 (18)	2 (9)
**Specimens from animals**		
No. received	60	7
No. laboratory-confirmed as *Dracunculus medinensis* (%)	53 (88)	5 (71)
**Country/Species of origin, no. of specimens (no. of animals)**		
**Angola**	—	3
Dog	—	3 (1)
**Chad**	8	—
Cat	2 (2)	—
Dog	6 (6)	—
**Ethiopia**	25	1
Baboon	6 (1)	1 (1)
Cat	6 (5)	—
Dog	13 (11)	—
**Mali**	20	1
Cat	2 (2)	—
Dog	18 (18)	1 (1)
**No. ruled out as *D. medinensis* (%)**	7 (12)	2 (29)
**No. of other laboratory diagnoses (%)**		
Ascarid	1 (14)	—
*Dirofilaria*	—	1 (50)
*Dipetalonema*	1 (14)	—
*Physaloptera*	1 (14)	—
Sparganum	1 (14)	—
Worm of unknown species	3 (43)	—
Unknown origin	—	1 (50)

* 19 worms from 16 patients in 2018 and two worms from one patient who had emerging worms in 2017.

^†^ 14 worms from 14 patients in 2019 and one worm from one patient in 2018.

^§^ One worm from one patient in 2017.

In affected countries, the national GWEP receives monthly reports of cases from supervised volunteers in each village under active surveillance** (Table 3). Villages where endemic transmission of dracunculiasis has ended (i.e., zero human cases or animal infections reported for ≥12 consecutive months) are kept under active surveillance for 2 additional years. WHO certifies a country as dracunculiasis-free after adequate nationwide surveillance for ≥3 consecutive years with no indigenous human cases or animal infections.^††^

**TABLE 3:** Reported human and animal dracunculiasis cases, active surveillance, and status of local interventions in villages with endemic disease, by country — worldwide, 2018

Human cases/Surveillance/Intervention status	Country					Total
	Chad*	Ethiopia	Mali^†^	South Sudan	Angola	
**Reported human cases**						
No. indigenous, 2018	17	0	0	10	1	28
No. imported,^§^ 2018	0	0	0	0	0	0
% contained^¶^ in 2018	41	0	0	30	0	36
% change in indigenous human cases in villages/localities under surveillance, same period, 2017 and 2018	+13	−100	0	NA	NA	−7
**Reported animal cases**						
No. indigenous, 2018	1,065	17	13	0	0	1,095
No. imported,** 2018	0	0	7	0	0	7
% contained^¶^ in 2018	75	41	80	0	0	74
% change in indigenous animal cases in villages/localities under surveillance, same period, 2017 and 2018	+28	+13	+100	0	0	+29
**Villages under active surveillance, 2018**						
No. of villages	1,895	156	903	2,121	0	5,075
% reporting monthly	99	100	99	92	0	96
No. reporting ≥1 human case	11	0	0	10	1	22
No. reporting only imported** human cases	0	0	0	0	1	1
No. reporting indigenous human cases	11	0	0	10	0	21
No. reporting ≥1 animal case	335	8	18	0	0	361
No. reporting only imported** animal cases	0	0	3	0	0	3
No. reporting indigenous animal cases	335	8	12	0	0	355
**Status of interventions in villages with endemic human dracunculiasis, 2018**						
No. of villages with endemic human dracunculiasis, 2017–2018	24	1	—	10	1	36
% reporting monthly^††^	100	100	—	100	—	100
% with filters in all households^††^	100	100	—	100	—	100
% using temephos^††^	55	100	—	100	—	67
% with ≥1 source of safe water^††^	71	0	—	50	100	64
% provided health education^††^	100	100	—	100	100	100
**Status of interventions in villages with endemic animal dracunculiasis, 2018**						
No. of villages with endemic animal dracunculiasis, 2017–2018	442	10	15	—	—	467
% reporting monthly^††^	100	100	100	—	—	100
% using temephos^††^	24	100	100	—	—	28
% provided health education^††^	100	100	100	—	—	100

**Abbreviation:** NA = not applicable.

* Participants at the annual Chad Guinea Worm Eradication Program review meeting in November 2014 adopted “1+ case village” as a new description for villages in Chad affected by human cases of Guinea worm disease and/or dogs infected with Guinea worms and defined it as “a village with one or more indigenous and/or imported cases of Guinea worm infections in humans, dogs, and/or cats in the current calendar year and/or previous year.”

^†^ Civil unrest and insecurity resulting from a coup d’état in 2012 continued to constrain Guinea Worm Eradication Program operations (supervision, surveillance, and interventions) in Gao, Kidal, Mopti, Segou, and Timbuktu regions.

^§^ Imported from another country.

^¶^ Transmission from a patient with dracunculiasis is contained only if all of the following conditions are met for each emerging worm: 1) the infected patient is identified ≤24 hours after worm emergence; 2) the patient has not entered any water source since the worm emerged; 3) a village volunteer or other health care provider has managed the patient properly, by cleaning and bandaging the lesion until the worm has been fully removed manually and by providing health education to discourage the patient from contaminating any water source (if two or more emerging worms are present, transmission is not contained until the last worm is removed); 4) the containment process, including verification of dracunculiasis, is validated by a Guinea Worm Eradication Program supervisor within 7 days of emergence of the worm; and 5) temephos is used to treat potentially contaminated surface water if any uncertainty about contamination of these sources of drinking water exists, or if a such a source of drinking water is known to have been contaminated.

** Imported from another in-country village with endemic disease.

^††^ The denominator is the number of villages/localities where the program applied interventions during 2017–2018.

## Country Reports

**Angola.** Before 2018, no case of dracunculiasis was ever reported from Angola. Following the discovery of a case in a girl with no history of foreign travel in Cunene Province in April 2018, Angolan health authorities and WHO investigated, searched nearby communities, and began training local health professionals and community health workers about the disease (*4*), but found no other active cases. Another case in a person with no history of foreign travel was detected in January 2019, and in April 2019 a dog with an emerging Guinea worm was found in the same district as the first case. Provisional DNA analysis of Angola’s Guinea worm specimens yielded no clear link to another *D. medinensis* population.

**Chad**. Chad reported 17 cases in 11 villages in 2018. During the first half of 2019, Chad reported 23 cases in 11 villages, compared with four cases reported during the first half of 2018 (Table 1). Twelve of the 23 cases reported in January–June 2019 were associated with one village in Salamat Region, in Chad’s first apparent waterborne outbreak of dracunculiasis in humans since 2010. A Cameroonian woman had a Guinea worm emerge in March 2019 in a village about one mile (1.5 km) from the Chad-Cameroon border; she was likely infected in Chad.

During 2018, 1,040 domestic dog and 25 domestic cat infections were reported, significantly more than the 817 dog and 13 cat infections reported in 2017 (Table 1). During January–June 2019, 93% more infected dogs and 20% more infected cats were reported than were reported during January–June 2018. The Carter Center is helping the Chad Ministry of Health implement active village-based surveillance for animal and human infections in 2,138 at-risk villages (as of June 2019), a 12% increase from 1,895 villages in December 2018. Based on previous investigations, the working hypothesis is that humans and dogs might become infected without drinking contaminated water, perhaps by eating inadequately cooked fish or other aquatic transport or paratenic hosts (intermediate hosts in which the parasite does not develop) (*7*). Since June 2017, approximately 81% of households sampled monthly in at-risk communities were burying fish entrails according to recommendations. Seventy-five percent of infected dogs were tethered (contained) in 2018 and 79% in January–June 2019. Temephos application to kill copepod intermediate hosts of the parasite in water reached 24% of 334 villages with dog or human infections as of December 2018 and 79% of villages by May 2019. In December 2018, 71% of villages reporting infected dogs or humans had at least one source of drinking water free from copepods.

In areas under surveillance in Chad, 85% of residents surveyed in 2018 knew of the cash rewards for reporting a human or animal infection, and 59% of those surveyed during January–June 2019 knew of the rewards. Intensified surveillance generated 41,501 rumors of infections in dogs and humans during January–June 2019, compared with 9,287 rumored infections during January–June 2018 (a rumor is a report of any information about a possible Guinea worm infection; a person or dog with compatible signs or symptoms is suspected of having dracunculiasis, pending confirmation).

**Ethiopia**. Ethiopia reported no human dracunculiasis cases during January 2018–June 2019 (Table 1). During 2018, Ethiopia reported 17 infected animals, including 11 dogs, five cats, and one baboon, all in Gog district of Gambella Region, compared with 15 infected animals (11 dogs and four baboons) in 2017. During January–June 2019, Ethiopia reported no infected dogs or cats, but six infected baboons, all in Gog district, compared with eight infected dogs and two infected cats during January–June 2018.

Since 2017, The Carter Center has supported Ethiopian public health and wildlife authorities in a baboon and dog epidemiology project. The project examined 28 live-captured baboons in January 2019, and none were found to have signs of Guinea worm infection. In June 2019, two of 33 trapped and released baboons were discovered with unemerged Guinea worms and two others with emergent Guinea worms; during the same month, villagers discovered two dead infected baboons, one with emergent Guinea worms and one with unemerged Guinea worms.

The Ethiopia Dracunculiasis Eradication Program (EDEP) has 156 villages under active surveillance. It applied temephos monthly to almost all water sources known to have been used by humans in the at-risk area of Gog district in 2015 and increased coverage to include numerous smaller water sources during 2016–2018. Since April 2018, EDEP has supported villager-initiated, proactive, constant tethering of approximately 1,100 dogs and cats in villages where most infected animals were detected in recent years to prevent their exposure to water sources in adjacent forests where transmission is believed to occur. Enhanced support now includes providing food, shelter, water, veterinary care, and daily exercise for the tethered animals. Ethiopia increased its reward for reporting a human dracunculiasis case from the equivalent of US$100 to US$360 in 2018 and increased the reward for reporting and tethering an infected animal from US$20 to US$40. In 2018, 81% of persons surveyed in areas under active surveillance were aware of the rewards.

**Mali.** In 2018, Mali reported no human dracunculiasis case for the third successive year, and no case during January–June 2019. During 2018, 18 infected dogs and two infected cats were reported, compared with nine dogs and one cat in 2017. During the first half of 2019, two infected dogs and no cats were reported, compared with three dogs and no cats during the first half of 2018 (Table 1). Twelve of the 20 infected animals identified in 2018 were detected in Segou Region; the remaining eight dogs were detected in adjacent Djenne District of Mopti Region. Segou Region is accessible to the program, but the dogs were bred and apparently became infected in areas of Mopti Region that have not been accessible to the program since 2012 because of insecurity; the dogs were later taken to Segou and sold for food. The two dogs reported during January–June 2019 were detected in Mopti Region near the presumed source of their infection, which still was not fully accessible to the program. The number of villages under active surveillance increased from 903 at the end of 2018 to 2,802 in 2019. In addition, the reward for reporting a human case was increased to the equivalent of US$340 (from US$100); the reward remains US$20 for reporting and tethering an infected animal. In areas under active surveillance, 80% of persons queried in 2018 were aware of the rewards for reporting an infected person or animal. A team from WHO conducted an external evaluation of Mali’s program in September–October 2018. They found no evidence of recent human infections and recommended improvements in preparation for precertification.

**South Sudan.** After reporting no cases of dracunculiasis for the first time in 2017, South Sudan reported 10 human cases in 2018. Eight patients were young cattle herders from migratory communities in recently pacified areas that had experienced chronic communal violence and population displacements in recent years. Extreme mobility of cattle herders and others is a special challenge in addition to sporadic insecurity. South Sudan reported no cases in January–June 2019, compared with four cases in January–June 2018 (Table 1). Only one infected animal has ever been reported: a dog in the same household as an infected person in 2015. By December 2018, South Sudan’s GWEP had 2,165 villages under active surveillance. In January 2018, the Ministry of Health increased the reward for reporting a case of dracunculiasis to the equivalent of approximately US$400, from US$140. A 2018 survey of 1,694 residents in villages under active surveillance found 72% of the respondents knew of the reward for reporting an infected person.

## Discussion

During January 2018–June 2019, Chad reported approximately 95% of the world’s *D. medinensis* infections, 96% of which were in dogs. After a decade with no reported cases, Chad reported 10 indigenous cases in humans in 2010, and Guinea worm infections in domestic dogs were reported for the first time in 2012, all primarily from communities along the Chari River (*7*). Stopping transmission among dogs in Chad is now the biggest challenge faced by the eradication program, which is being addressed through expanded and innovative interventions, using field and laboratory research supported by The Carter Center and CDC to better understand the unusual epidemiology of dracunculiasis in Chad and assess antihelminthic treatment of dogs to prevent maturation of worms (*8*). In collaboration with researchers from the University of Georgia (Athens, Georgia), this initiative has shown that fish can serve as transport hosts for *Dracunculus* spp. in the laboratory and that *D. medinensis* can use frogs as paratenic hosts; *Dracunculus* larvae have been recovered from multiple wild frogs in Chad (*9*,*10*).

Before 2010, Chad’s ministry of health began offering a reward equivalent to US$100 for reporting a confirmed human dracunculiasis case, and the program introduced a reward of US$20 in February 2015 for reporting and tethering an infected dog. The rewards are given only after a case is confirmed; all reports must be corroborated by supervisors. In 2017, Chad launched a nationwide communication campaign to increase awareness of the cash rewards and knowledge about how to prevent Guinea worm infections in humans and dogs. Since October 2013, Chad’s GWEP urged villagers to cook their fish well, bury fish entrails, and prevent animals from eating them. In February 2014, health educators also began persuading villagers to tether infected dogs until the worms emerged to prevent contamination of water. Because water treatment with temephos is constrained by the extremely large lagoons used for fishing and as sources of drinking water, application of temephos to cordoned sections of the lagoons at entry points used by infected humans or dogs was introduced to protect villages in 2014. In October 2017, monthly temephos applications began at small ponds in villages with the most infected dogs.

The pattern of transmission to many dogs and few humans in Chad remains peculiar to that country. If the hypothesis that the parasite’s life cycle in Chad involves a transport or paratenic host (*10*) is correct, increased active surveillance, containment of infected dogs, application of temephos, and burial of fish entrails should reduce transmission. The dracunculiasis case found in a Cameroonian border village in April 2019 highlights the risks for cases exported from Chad and the need for ongoing active surveillance in neighboring countries, especially Cameroon and Central African Republic.

The surprising discovery of dracunculiasis in Angola is worrisome. Finding only two confirmed cases in humans and one infected dog in one Angolan province to date in 2018–2019 suggests that the problem there is limited, but active surveillance throughout the areas at risk is required to determine its full extent. South Sudan appears poised to recover its zero-case status quickly with strong technical leadership, strong political support by the government, and without parallel infections in animals, if adequate security can be maintained.

As of June 2019, Mali and Ethiopia had not reported dracunculiasis in a human in 3.5 and 1.5 consecutive years, respectively. Continued endemic transmission of Guinea worm infections among a few dogs and cats in Mali as well as baboons in Ethiopia appears to be geographically limited in each country. The ecologic study of baboons and proactive tethering of dogs in Gog district might help elucidate the unusual dynamics of residual Guinea worm infections in Ethiopia. Insecurity has decreased in some areas of Mali with endemic transmission in 2019 but is still the main obstacle to stopping transmission among dogs in that country. DNA studies show promise for tracing genetic lineages of worms, which will provide another tool for understanding *D. medinensis* transmission dynamics.

**Summary**

**What is already known about this topic?**

The number of cases of dracunculiasis (Guinea worm disease) has decreased from an estimated 3.5 million in 1986 to 28 in 2018. Emergence of Guinea worm infections in dogs has complicated eradication efforts.

**What is added by this report?**

During January–June 2019, the number of human dracunculiasis cases reported increased to 25 cases in three countries (Angola, Cameroon, and Chad) and 1,346 infected domestic dogs were reported; Ethiopia, Mali, and South Sudan reported no human cases.

**What are the implications for public health practice?**

Existence of infected dogs, especially in Chad, and impeded access because of civil unrest and insecurity in Mali and South Sudan are now the greatest challenges to interrupting transmission.

## Footnotes

* Transmission from a patient with dracunculiasis is contained only if all of the following conditions are met for each emerging worm: 1) the infected patient is identified ≤24 hours after worm emergence; 2) the patient has not entered any water source since the worm emerged; 3) a village volunteer or other health care provider has managed the patient properly, by cleaning and bandaging the lesion until the worm has been fully removed manually and by providing health education to discourage the patient from contaminating any water source (if two or more emerging worms are present, transmission is not contained until the last worm is removed); 4) the containment process, including verification of dracunculiasis, is validated by a Guinea Worm Eradication Program supervisor within 7 days of emergence of the worm; and 5) the approved chemical temephos (Abate) is used to treat potentially contaminated surface water if any uncertainty about contamination of the source of drinking water exists, or if such a source of drinking water is known to have been contaminated. Similar criteria are in place for the containment of animal infections.

† A dracunculiasis case is defined as an infection occurring in a person exhibiting a skin lesion or lesions with emergence of one or more worms laboratory-confirmed at CDC as *D. medinensis*. Because *D. medinensis* has a 10- to 14-month incubation period, each infected person is counted as having a case only once during a calendar year.

§ Initially 20 countries, but the former country of Sudan officially separated into two countries (Sudan and South Sudan) on July 9, 2011.

¶ Specimens are laboratory-confirmed as *D. medinensis* at CDC by either morphologic examination under a microscope or polymerase chain reaction assay. Additional information about laboratory identification of parasites is available at https://www.cdc.gov/dpdx/dxassistance.html.

** Villages under active surveillance are those that have endemic dracunculiasis or are at high risk for importation. Active surveillance involves daily searches of households by village volunteers (supported by their supervisors) for persons or animals with signs of dracunculiasis. An imported human case or animal infection is one resulting from ingestion of contaminated water in a place other than the community where the case or infection is detected and reported. Since 2012, no internationally imported cases or infections have been reported.

†† An indigenous dracunculiasis human case or animal infection is defined as an infection consisting of a skin lesion or lesions with emergence of one or more Guinea worms in a person or animal who had no history of travel outside their residential locality during the preceding year.
