# *Erratum:* Vol. 68, No. 43

**DOI:** 10.15585/mmwr.mm6847a3

**Published:** 2019-11-29

In the report “Progress Toward Global Eradication of Dracunculiasis — January 2018–June 2019,” on page 979, a sentence was omitted from the first paragraph. The paragraph should have read as follows:

“Dracunculiasis (also known as Guinea worm disease) is caused by the parasite *Dracunculus medinensis* and is acquired by drinking water containing copepods (water fleas) infected with *D. medinensis* larvae. The worm typically emerges through the skin on a lower limb approximately 1 year after infection, resulting in pain and disability (*1*). There is no vaccine or medicine to treat the disease; eradication efforts rely on case containment* to prevent water contamination and other interventions to prevent infection, including health education, water filtration, chemical treatment of unsafe water with temephos (an organophosphate larvicide to kill copepods), and provision of safe drinking water (*1*,*2*). **The worldwide eradication campaign began in 1980 at CDC**. In 1986, with an estimated 3.5 million cases^†^ occurring each year in 20 African and Asian countries^§^ (*3*), the World Health Assembly called for dracunculiasis elimination (*4*). The global Guinea Worm Eradication Program (GWEP), led by The Carter Center and supported by the World Health Organization (WHO), CDC, the United Nations Children’s Fund, and other partners, began assisting ministries of health in countries with dracunculiasis. This report, based on updated health ministry data, describes progress to eradicate dracunculiasis during January 2018–June 2019 and updates previous reports (*2*,*4*,*5*). With only five countries currently affected by dracunculiasis (Angola, Chad, Ethiopia, Mali, and South Sudan), achievement of eradication is within reach, but it is challenged by civil unrest, insecurity, and lingering epidemiologic and zoologic questions.”

